# Vitamin C alters the amount of specific endoplasmic reticulum associated proteins involved in lipid metabolism in the liver of mice synthesizing a nonfunctional Werner syndrome (Wrn) mutant protein

**DOI:** 10.1371/journal.pone.0193170

**Published:** 2018-03-01

**Authors:** Lucie Aumailley, Florence Roux-Dalvai, Isabelle Kelly, Arnaud Droit, Michel Lebel

**Affiliations:** 1 Centre de recherche du CHU de Québec, Faculty of Medicine, Université Laval, Quebec City Québec, Canada; 2 Proteomics Platform Center, Centre de recherche du CHU de Québec, Faculty of Medicine, Université Laval, Quebec City Québec, Canada; Chinese University of Hong Kong, HONG KONG

## Abstract

Werner syndrome (WS) is a premature aging disorder caused by mutations in a protein containing both a DNA exonuclease and DNA helicase domain. Mice lacking the helicase domain of the Wrn protein orthologue exhibit transcriptomic and metabolic alterations, some of which are reversed by vitamin C. Recent studies on these animals indicated that the mutant protein is associated with enriched endoplasmic reticulum (ER) fractions of tissues resulting in an ER stress response. In this study, we identified proteins that exhibit actual level differences in the ER enriched fraction between the liver of wild type and Wrn mutant mice using quantitative proteomic profiling with label-free Liquid Chromatography-Tandem Mass Spectrometry (LC-MS/MS). Multiple Reaction Monitoring (MRM) and immunoblotting were performed to validate findings in a secondary independent cohort of wild type and Wrn mutant mice. DAVID 6.7 (NIH) was used for functional annotation analysis and indicated that the identified proteins exhibiting level changes between untreated wild type, Wrn mutant, and vitamin C treated Wrn mutant mice (ANOVA *P*–value < 0.05) were involved in fatty acid and steroid metabolism pathways (Bonferroni *P*-value = 0.0137). Finally, when we compared the transcriptomic and the proteomic data of our mouse cohorts only ~7% of the altered mRNA profiles encoding for ER gene products were consistent with their corresponding protein profiles measured by the label-free quantification methods. These results suggest that a great number of ER gene products are regulated at the post-transcriptional level in the liver of Wrn mutant mice exhibiting an ER stress response.

## Introduction

Werner syndrome (WS) is a human autosomal recessive disorder characterized by genomic instability and the premature onset of a number of age-related diseases [1–3]. The defective enzyme responsible for WS possesses a 3**′**-5**′** exonuclease activity in addition to a 3**′**-5′ DNA helicase activity [4, 5] and is involved in DNA repair, replication, transcription, and telomere maintenance [6–10]. We previously generated a mouse model with a deletion in the helicase domain of the murine WRN orthologue [11] that recapitulates many of the WS phenotypes, including an abnormal hyaluronic acid excretion, dyslipidemia, insulin resistance, and increased cancer incidence [12, 13]. Overall, such mutant mice have a 17–22% decreased in their mean life span compared to wild type animals [14, 15]. Interestingly, the treatment of these Wrn mutant mice (referred as *Wrn*^*Δhel/Δhel*^ hereafter) with vitamin C extended the life span of such animals to the normal wild type phenotype [14, 16]. In addition to the metabolic abnormalities found in *Wrn*^*Δhel/Δhel*^ mice [15, 16], microscopic analysis of tissues revealed premature de-fenestration of liver sinusoidal endothelial cells and hepatic steatosis [17, 18], aortic stenosis [19] followed by cardiac fibrosis [13]. Notably, dyslipidemia is one of the first major phenotypes observed in four to five-month old mutant mice [13] in addition to changes in inflammatory cytokines and cardiometabolic risk factors [15, 16]. Such phenotypes were reversed by vitamin C treatment. Finally, microarray and gene set enrichment analyses on the liver tissues of nine-month old *Wrn*^*Δhel/Δhel*^ mice revealed that vitamin C treatments decreased genes normally up-regulated in human WS fibroblasts and cancers, and it increased genes involved in tissue injury response and adipocyte dedifferentiation in obese mice [14].

*Wrn*^*Δhel/Δhel*^ mice synthesize a stable mutant protein in tissues. Remarkably, fractionation experiments have indicated that the Wrn mutant helicase protein is associated with the endoplasmic reticulum (ER) and the peroxisomal fractions of liver tissues [15]. Furthermore, mRNA expression analyses indicated that the helicase mutation altered the expression of many genes coding for proteins associated with the ER in the liver of *Wrn*^*Δhel/Δhel*^ mice [14]. However, changes at the protein level of such genes coding for ER-associated proteins have never been confirmed in these mice. Recent innovations in proteomic technologies allow the identification and quantification of thousands of proteins between samples [20]. In this study, we used high-throughput proteomic methodologies to specifically identify the alterations in the proteome profile of ER enriched fractions from the liver of *Wrn*^*Δhel/Δhel*^ mice treated with or without vitamin C compared to age-matched wild type animals.

## Materials and methods

### Animal model

Mice lacking part of the helicase domain of the *Wrn* gene were generated by homologous recombination, as described previously [11]. This study was performed on wild type and *Wrn*^*Δhel/Δhel*^ homozygous animals on C57BL/6NHsd genetic background (Harlan Laboratories, Frederick, MD) and was carried out in strict accordance with the recommendations in the Guide for the Care and Use of Laboratory Animals of the Canadian Council on Animal Care in science. The protocol was approved by the Committee on the Ethics and Protection of Animal of Laval University (Permit Number: 2014029). Mice were housed in microisolator cages (containing a top filter) at 22 ± 2°C with 40%–50% humidity and a 12-h light–dark cycle (light cycle: 06:00–18:00 hours) in the Centre Hospitalier de l’Université Laval animal facility. All mice were fed ad libitum with Teklad Global (cat. # 2018; Madison, WI) 18% protein rodent diet (5% fat). When indicated *Wrn*^*Δhel/Δhel*^ mice were treated with 0.4% L-ascorbate (w/v) in drinking water from weaning (21-day old) until the age of five months, age at which mice were euthanized for mass spectrometry and immunoblot analyses of the liver endoplasmic reticulum enriched fraction. Euthanasia was performed by treating mice with 3% isoflurane (general anesthesia) followed by cervical dislocation.

### Protein extraction from tissues and endoplasmic reticulum fractionation

Total endoplasmic reticulum (ER) fraction from mouse livers was obtained using an endoplasmic reticulum enrichment assay kit (Novus Biologicals, Burlington, ON, Canada) according to the manufacturer's protocol. Briefly, weighed and washed liver tissues were homogenized in isosmotic homogenization buffer containing phosphatase inhibitor cocktail (PhosphoSTOP EASYpack) from Roche Applied Science (Indianapolis, IN) and protease inhibitor cocktail provided by the kit. An aliquot of the homogenate (whole cell liver fraction H) was kept for western blot analysis. The homogenate was centrifuged at 1,000 *g* for 10 min at 4°C. The supernatant was recentrifuged at 12,000 *g* for 15 min at 4°C. The pellet was dissolved for 1 hour at 4°C in RIPA buffer (50 mM Tris-HCl (pH 7.5), 150 mM NaCl, 1% NP-40, 0.2% SDS, 1% sodium deoxycholate, 20 mM sodium pyrophosphate, 1 mM sodium orthovanadate, 1 mM phenylmethylsulfonylfluoride, complete protease inhibitor cocktail and PhosphoSTOP EASYpack from Roche Applied Science, Indianapolis, IN) to obtain a pellet fraction (P). The supernatant was recentrifuged at 90,000 *g* for 1 hour at 4°C. The new supernatant (fraction S) was frozen for western analysis. The pellet (ER fraction) obtained at the end of the procedure was finally disolved using the suspension buffer provided by the kit with phosphatase inhibitor cocktail (PhosphoSTOP EASYpack) and protease inhibitors. Protein concentration was determined by the Bradford protein assay (Bio-Rad, Mississauga, ON, Canada). Samples were frozen at -80°C until mass spectrometry or immunoblotting analyses.

### Solubilisation and in-solution digestion of proteins from ER fractions for mass spectrometry analyses

Equal amounts of protein (50 μg) were solubilised in the denaturation buffer (ammonium bicarbonate 50 mM (pH8)—sodium deoxycholate 1%, 30μl final volume). Then samples were heated to 95°C for 5 min. Disulfide bonds were reduced with 1μg DTT (30 min at 37°C) and alkylated with 5 μg iodoacetamide (30 min at 37°C in the dark). Finally, trypsin (1:50 w/w enzyme-to-protein ratio) was added and the mixture was incubated at 37°C for 24 h. The digestion was stopped by acidifying the sample to pH < 2.5 with 30 μL of 3% acetonitrile—0.1% trifluoroacetic acid—0.5% acetic acid solution. The precipitated sodium deoxycholate was eliminated by 10 min incubation at room temperature (RT) and centrifugation for 5 min (16,000 *g*) at RT. The supernatant was desalted on C18 Empore filter. Peptides were eluted in 80% acetonitrile– 0.1% formic acid and dried in a speed vacuum. The samples were reconstituted with 50 μL 0.1% formic acid. This tryptic digestion protocol was used for both the Label-Free Quantification and the Multiple Reaction Monitoring methods.

### Mass spectrometry

One μg of each sample was analyzed by NanoLC-MS/MS. Peptides resulting from trypsin digestion were injected and separated by online reversed-phase (RP) nanoscale capillary liquid chromatography (nanoLC) and analyzed by electrospray mass spectrometry (ESI MS/MS). The experiments were performed with a Dionex UltiMate 3000 nanoRSLC chromatography system (Thermo Fisher Scientific/Dionex Softron GmbH, Germering, Germany) connected to an Orbitrap Fusion mass spectrometer (Thermo Fisher Scientific, San Jose, CA, USA) equipped with a nanoelectrospray ion source. Peptides were trapped at 20 μL/min in loading solvent (2% acetonitrile, 0.05% trifluoroacetic acid) on a 5 mm x 300 μm C18 pepmap cartridge pre-column (Thermo Fisher Scientific/Dionex Softron GmbH, Germering, Germany) during 5 min. Then, the pre-column was switched online with a self-made 50 cm x 75 μm internal diameter separation column packed with ReproSil-Pur C18*-*AQ 3-μm resin (Dr. Maisch HPLC GmbH, Ammerbuch-Entringen, Germany) and the peptides were eluted with a linear gradient from 5–40% solvent B (A: 0.1% formic acid, B: 80% acetonitrile, 0.1% formic acid) for 150 min at 300 nL/min. Mass spectra were acquired using a data dependent acquisition mode using Thermo XCalibur software version 3.0.63. Full scan mass spectra (350 to 1800 m/z) were acquired in the orbitrap using an AGC target of 4e5, a maximum injection time of 50 ms and a resolution of 120,000. Internal calibration using lock mass on the m/z 445.12003 siloxane ion was used. Each MS scan was followed by acquisition of fragmentation MS/MS spectra of the most intense ions for a total cycle time of 3 s (top speed mode). The selected ions were isolated using the quadrupole analyzer in a window of 1.6 m/z and fragmented by Higher energy Collision-induced Dissociation (HCD) with 35% of collision energy. The resulting fragments were detected by the linear ion trap in rapid scan rate with an AGC target of 1e4 and a maximum injection time of 50 ms. Dynamic exclusion of previously fragmented peptides was set for a period of 20 s and a tolerance of 10 ppm. The mass spectrometry proteomics data have been deposited to the ProteomeXchange Consortium via the PRIDE [21] partner repository with the dataset identifier PXD007758.

### Database searching and Label Free Quantification

Spectra were searched against a mouse proteins database (Uniprot Complete Proteome–taxonomy Mus musculus– 43640 sequences) using the Andromeda module of MaxQuant software v. 1.5.2.8 [22]. Trypsin/P enzyme parameter was selected with two possible missed cleavages. Carbamidomethylation of cysteine was set as fixed modification, methionine oxidation, acetylation of protein N-terminus and phosphorylation on serine, threonine and tyrosine as variable modifications. Search mass tolerances were defined at 5 ppm and 0.6 Da for MS and MS/MS, respectively. For protein validation, a maximum False Discovery Rate of 1% at peptide and protein level was used based on a target/decoy search. MaxQuant was also used for Label Free Quantification. The ‘match between runs’ option was used with 20 min value as alignment time window and 3 min as match time window. The intensity values of ‘unique and razor’ peptides extracted by MaxQuant were normalized by the median for each protein in each sample replicate and used for quantification. When intensity values were missing, they were imputated with a noise value corresponding to the first percentile of all peptide intensity values of the sample replicate. Only peptides having at least two intensity values over the three replicates in one of the two conditions to compare, before missing values imputation, were considered for quantification. Then, all peptides intensities belonging to the same leading razor protein given by MaxQuant were summed together to get protein intensity values, which were then used to calculate the protein ratio between the two groups of samples to compare as well as Z-scores and statistical *P*-values as described in the section entitled statistical analyses below.

### Multiple Reaction Monitoring (MRM)

#### Peptide selection and stable-isotope-labeled standard (SIS) peptides

In order to select tryptic peptides that are the most suitable for sensitive and selective protein detection, MRM analyses were done on tryptic peptides that were detected in previous mass spectrometry analyses with length ranging from 5 to 25 amino acids [23]. Peptides containing methionine and missed trypsin cleavage were eliminated. Peptides were selected based on peak shape and intensity. Crude synthetic peptides (PEPotec) containing [^13^C_6_]-Lys and [^13^C_6_]-Arg were obtained from ThermoFisher Scientific (Germany). The peptides were diluted and pooled to a final concentration ranging from 0.1 to 100 pmol/μL. One μL of the solution was spiked into 9 μL of reconstituted sample solution after tryptic digestion.

#### LC-MRM analysis

One μg of peptides (in 2 μL) were analyzed on a ABSciex 6500QTRAP^TM^ hybrid triple quadrupole/linear ion trap mass spectrometer equipped with an Eksigent nanoLC AS2 controlled by Analyst 1.6^TM^ and with a nanospray ionization source. MS analysis was conducted in positive ion mode with an ion spray voltage of 2300 V. Peptides were desalted on a 200 μm x 0.5 mm trap column packed with ChromXP C18 3 μm resin (Eksigent, Dublin, CA) at 4 μL/min of Solvent A (0.1% formic acid) then switched in line at a flow rate of 300 nL/min on a 75 μm x 15 cm column packed with ChromXP C18 3 μm resin (Eksigent) with a 28 min linear gradient from 5 to 40% of solvent B (acetonitrile– 0.1% formic acid), then a 10 min linear gradient from 40 to 95% of solvent B, followed by a 17 min linear gradient. Cycle times were 2.9 s. Nebulizer gas was set to 8 (Gas1), curtain gas to 20, heater to 150°C and declustering potential (DP) to 70 V. Scheduled LC-MRM analyses were performed using three transitions on one to two peptides for each of the target proteins. The quantification was done with Skyline 3.6 and was based on the relative areas of the SIS and endogenous peptides. The areas of MRM transitions for each peptide were summed in order to calculate the area ratio. A blank solvent injection was run between biological samples to prevent sample carry over on the HPLC column. Samples were injected in random order.

### Immunoblotting analysis

Protein samples were resolved using 8 or 10% sodium dodecyl sulfate-polyacrylamide gel electrophoresis and then transferred onto 0.2 μm polyvinylidene difluoride membrane (EMD Millipore Corporation, Temecula, CA). After incubating 1 h with blocking solution (PBS-T or TBS-T containing 5% nonfat milk), the membrane was probed overnight at 4°C with a primary antibody. After washing with PBS-T or TBS-T, species-specific horseradish peroxidase-conjugated secondary antibody was added for 2 h at room temperature (GE Healthcare Limited, Piscataway, NJ). Signals were generated with ECL reagents from Amersham Biosciences (Piscataway, NJ). When indicated, immunoblots were probed with the following antibodies: rabbit polyclonal antibodies raised against the eukaryotic translation initiation factor 2-α kinase 3 (anti-PERK (H300): sc-13073), eukaryotic translation factor 2-α (anti-eIF2α (FL-315): sc-11386), phosphorylated eIF2α (anti-p-eIF2α (Ser 52): sc101670), heat shock cognate 70 kDa protein (anti-HSP70/HSC70 (H300): sc-33575), fatty acid synthase (anti-FASN (H-300): sc-20140) and sodium dependent vitamin C transporter (anti-SVCT1 (H-78): sc-30113) from Santa Cruz Biotechnology (Santa Cruz, CA); a rabbit monoclonal antibody against inositol-requiring kinase 1α (anti-IRE1 (14C10) #3294) from Cell Signaling Technology (Beverly, MA); a rabbit polyclonal antibody against glucose-related protein 78 (anti-GRP78) from Proteintech^TM^ (Chicago, IL); rabbit polyclonal antibodies against calreticulin (anti-calreticulin–ER marker ab39818), and cysteine sulfinic acid decarboxylase (anti-Csad antibody ab91016) from Abcam (Cambridge, MA); rabbit monoclonal antibodies against epidermal growth factor receptor (anti-EGFR antibody [EPR39Y] ab76153) and topoisomerase I (anti-topoisomerase I [EPR5375] ab109374) from Abcam (Cambridge, MA); a rabbit polyclonal antibody against mitochondrial manganese superoxide dismutase (anti-MnSOD #06–984) from EMD Millipore Corporation (Temecula, CA) and mouse monoclonal antibodies against β-actin (A5441) and catalase (C0979) from Sigma-Aldrich (Oakville, ON).

### Microarray analysis

Total RNA from the liver of three-month old WT, *Wrn*^*Δhel/Δhel*^, and ascorbate-treated (since weaning) *Wrn*^*Δhel/Δhel*^ mice was extracted on a CsCl cushion. RNAs were processed at the functional genomic platform of the Institut de Recherche Clinique de Montreal (Montreal, QC, Canada) and were hybridized onto mouse Agilent 60-mer Oligo Microarrays (44,000 genes/microarray; Agilent Technologies, Santa Clara, CA, USA) in quadruplicates with dye swap. Arrays were scanned on an Axon GenePix 4000B (Axon Instruments, Foster City, CA, USA). Data were extracted from images using GenePix 6.0 (Axon) with local background estimation. Lists of differentially expressed genes were generated using limma in BioConductor (http://www.bioconductor.org). The data were background subtracted using normexp and also normalized using the loess method. Correction for multiple hypothesis testing was performed using Benjamini-Hochberg. We have deposited all the raw data (accession number GSE66584 and GSE66642) in the U.S. National Center for Biotechnology Information public database Gene Expression Omnibus (http://www.ncbi.nlm.nih.gov/geo/).

### Statistical analyses

To identify proteins differentially expressed between two groups of mice, a Welch’s *t*-test was performed and the Z-score was calculated. The Z-score is the distance between the raw score and the population mean in units of the standard deviation. Differences were considered significant between two mouse cohorts when the Welch’s *t*-test *P*-value < 0.05 and the absolute Z-score > 2.0 (i.e. outside the 95% confidence interval). Finally, the identification of significantly expressed proteins between all three cohorts of mice was achieved by using the one-way ANOVA followed by Tukey’s HSD (honest significant difference) Test for post-ANOVA pair-wise comparisons on the LFQ intensities. These tests were calculated using the http://statistica.mooo.com/OneWay_Anova_with_TukeyHSD website. Differences were considered significant at a *P*-value < 0.05. Principal component analysis (PCA) and generation of heatmaps containing clustering dendrograms were generated using the http://biit.cs.ut.ee/clustvis/ website.

The PANTHER (Protein ANalysis THrough Evolutionary Relationships) classification system was used to classify proteins by their functions. This program is implemented in the DAVID (Database for Annotation, Visualization, and Integrated Discovery) web site [24]. Enrichments for specific biological functions using PANTHER were considered significant with a Bonferroni value smaller than 0.05.

## Results

### ER stress response in the liver of wild type and *Wrn*^*Δhel/Δhel*^ mice

**Fig 1:**
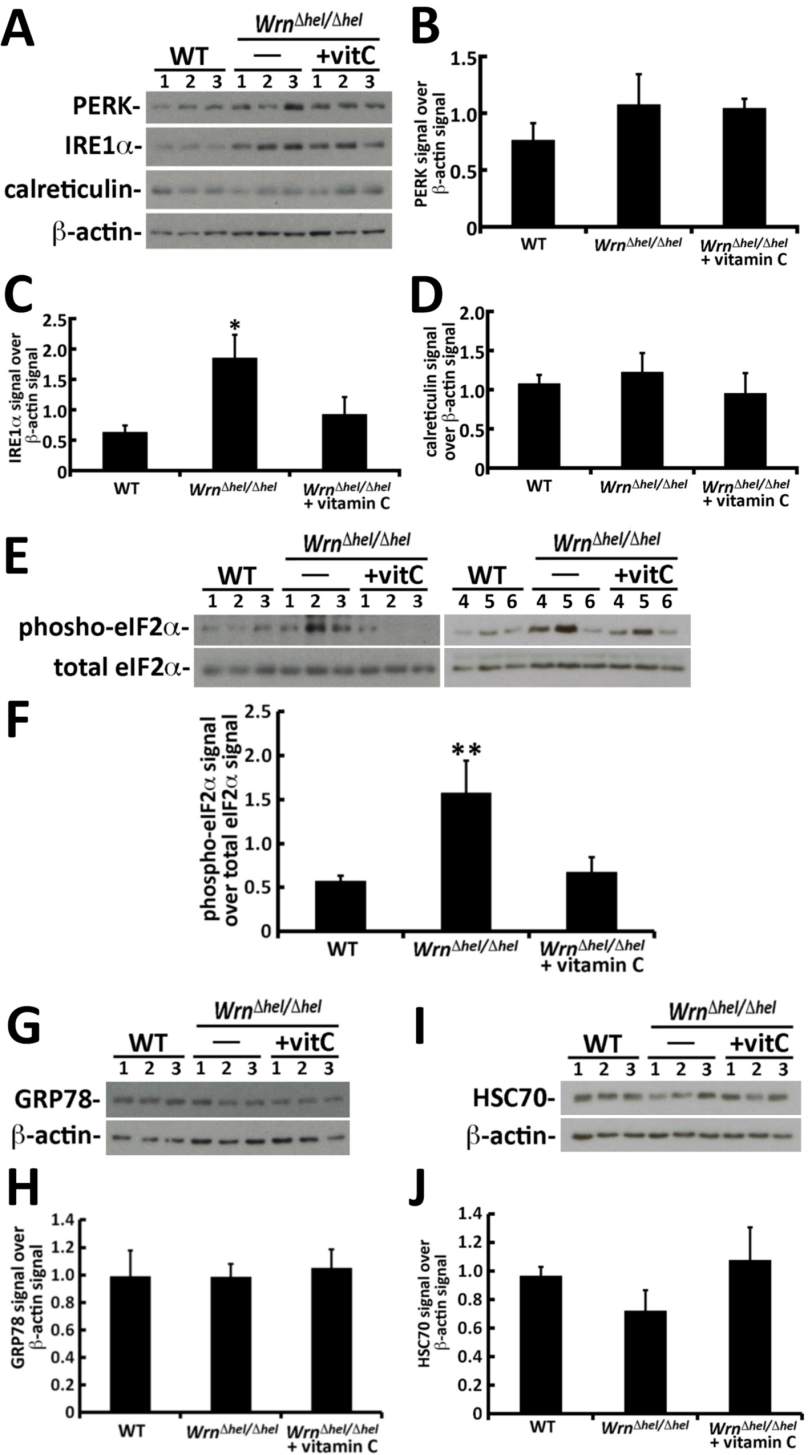
Impact of vitamin C on the levels of different ER stress markers in liver total lysates from *Wrn*^*Δhel/Δhel*^ mice. (A) Example of western blots showing protein levels of total PERK, total IRE1α, and calreticulin in three mice of each group. β-actin was used as loading controls. (B) Ratio of PERK signal over β-actin signal from the western blots. (C) Ratio of total IRE1α signal over β-actin signal from the western blots. (ANOVA: *P* > 0.05; but student *t*-test: *P = 0.039 for *Wrn*^*Δhel/Δhel*^ mice versus wild type mice). (D) Ratio of calreticulin signal over β-actin signal from the western blots. (E) Western blots showing protein levels of total eIF2α and its phosphorylated form in six animals of each group. (F) Ratio of phosphorylated eIF2α signal over total eIF2α signal. (Tukey post ANOVA test: **P = 0.016 compared to all other groups of mice). (G) Western blot showing protein levels of total GRP78 in three mice of each group. β-actin was used as loading controls. (H) Ratio of GRP78 signal over β-actin signal from the western blots. (I) Western blot showing protein levels of total HSC70 in three mice of each group. β-actin was used as loading controls. (J) Ratio of HSC70 signal over β-actin signal from the western blots. Bars in all histograms represent SEM.

We previously found a significant increase in reactive oxygen species (ROS) (~11%) in the ER enriched fraction of *Wrn*^*Δhel/Δhel*^ liver tissues compared to the liver ER enriched fraction of age-matched wild type animals [16]. This increase was reversed by the treatment of *Wrn*^*Δhel/Δhel*^ mice with 0.4% vitamin C (w/v) in drinking water [16]. We thus examined different ER stress markers in our WT and *Wrn*^*Δhel/Δhel*^ mice. PERK, IRE1α, eIF2α, and GRP78, were analyzed by western blotting. The chaperone HSC70, which is involved in the chaperon-mediated protein degradation pathway [25], was also examined in total liver extracts. β-actin was used as a loading control. Total PERK level was not significantly increased in the liver of *Wrn*^*Δhel/Δhel*^ mice compared to WT mice (Fig 1A and 1B). The level of phosphorylation was not significantly different between our three cohorts of mice (data not shown). Total IRE1α level was significantly increased in the liver of *Wrn*^*Δhel/Δhel*^ mice compared to WT animals (based on student’s *t*-test; *P* = 0.039). The difference between untreated and vitamin C treated *Wrn*^*Δhel/Δhel*^ mice was not statistically different (Fig 1C). Calreticulin level was not significantly different between groups of mice (Fig 1A and 1D). In contrast, the phosphorylation of eIF2α (a marker of ER stress) was significantly increased in the liver of *Wrn*^*Δhel/Δhel*^ mice. Vitamin C treatment significantly decreased this phosphorylation of eIF2α to WT level (Fig 1E and 1F; ANOVA *P*-value = 0.016). Total GRP78 level was not statistically different between groups of mice (Fig 1G and 1H). Finally, the chaperone HSC70 level was not significantly different between groups of mice. Although there were interindividual variations in the phosphorylation of eIF2α, the changes observed in total IRE1α and phosphorylated eIF2α levels suggest an activation of the ER stress response in *Wrn*^*Δhel/Δhel*^ mice compared to WT animals as previously described [15, 16].

### Identification of proteins in the liver ER enriched fractions of wild type and *Wrn*^*Δhel/Δhel*^ mice by mass spectrometry

**Fig 2:**
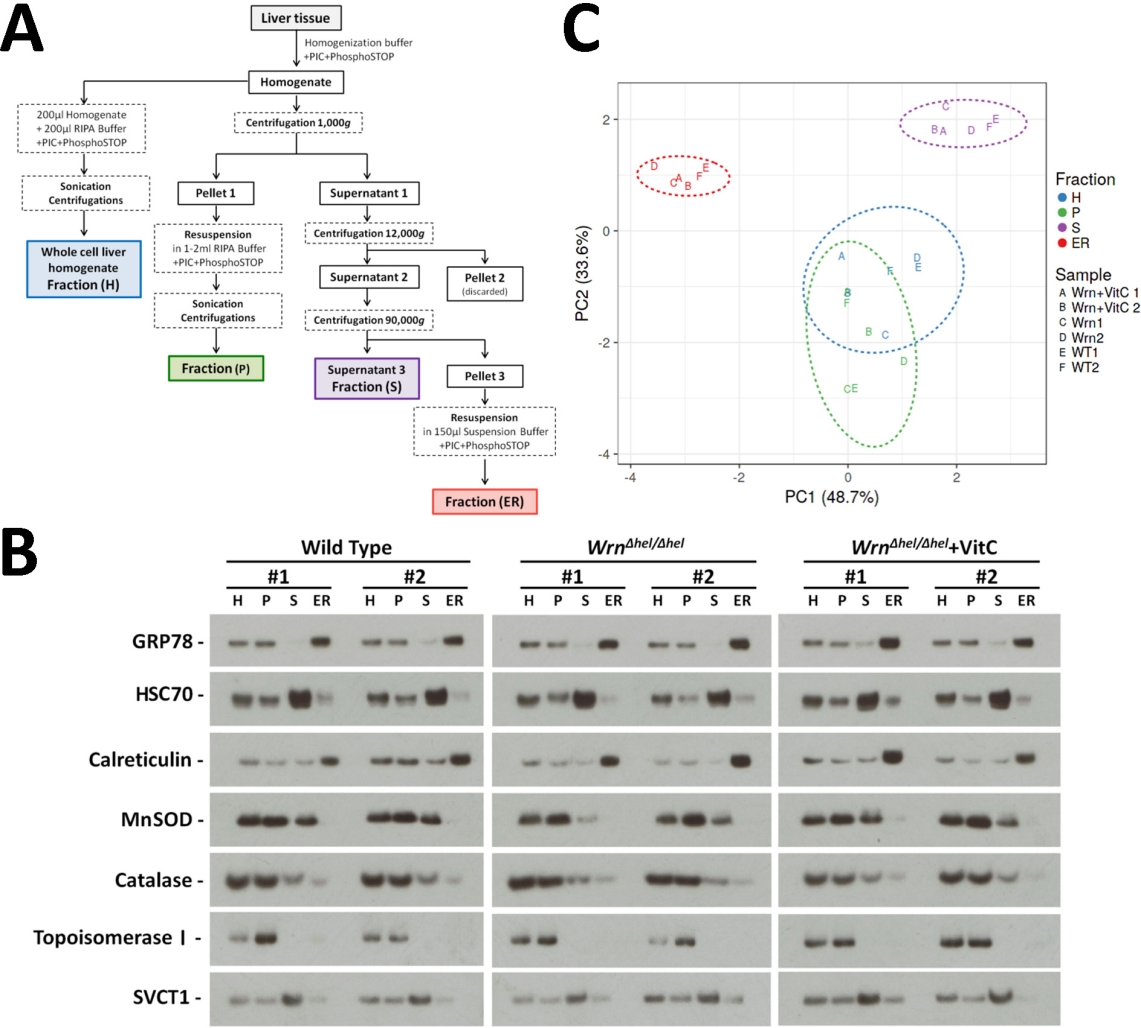
Efficiency of the liver ER enriched fractionation procedure on the different groups of mice. (A) Schematic representation of the different steps undertaken to obtain different cellular fractions. (B) Example of western blots showing protein levels of GRP78, HSC70, calreticulin, MnSOD, catalase, topoisomerase I, and SVCT1 in the different liver fractions. Each lane contains 15 μg of proteins. (H = whole cell homogenate; P = pellet fraction from the first step of the procedure; S = supernatant fraction of the last step of the procedure; ER = ER enriched fraction). (C) Principal component analysis (PCA) graph showing the consistency of the fractionation procedure when we measured the protein levels from the immunoblots shown in Fig 2B in the different fractions of vitamin C treated and untreated *Wrn*^*Δhel/Δhel*^ mice compared to wild type mice. WT = wild type mice; Wrn = *Wrn*^*Δhel/Δhel*^ mice; Wrn+VitC = *Wrn*^*Δhel/Δhel*^ mice treated with 0.4% vitamin C (w/v) in drinking water since weaning. X and Y axis show principal component 1 and principal component 2 that explain 48.7% and 33.6% of the total variance, respectively. Prediction ellipses are such that a new observation from the same group will fall inside the ellipse with a probability of 0.95.

To gain additional information on the biological processes that are altered in the ER of *Wrn*^*Δhel/Δhel*^ mice, we identified and quantified proteins from the ER enriched fractions of liver tissues from untreated wild type, *Wrn*^*Δhel/Δhel*^, and vitamin C treated *Wrn*^*Δhel/Δhel*^ mice by mass spectrometry analyses. We first estimated the efficiency of our ER fractionation procedure by monitoring the levels of specific proteins by western analyses at different steps of the enrichment procedure. A schematic of the different fractions that were analyzed during the procedure is presented in Fig 2A. The fractions that were analyzed by western blotting included the whole cell liver lysate (fraction H), the pellet fraction after the homogenization step (fraction P), the ER enriched fraction (ER), and the supernatant fraction of the last step (fraction S). The proteins that were analyzed are shown in the representative immunoblots from two different animals for each group of mice in Fig 2B. As expected, GRP78 was mainly found in the ER enriched fraction of the last step of the fractionation procedure. HSC70 was mostly found in the supernatant since it is mainly a cytoplasmic protein [26] but was also detected in the ER enriched fraction. Calreticulin (a protein found in both the nucleus and the lumen of the ER; https://www.ncbi.nlm.nih.gov/gene/811) gave the strongest signal in the ER enriched fraction. The mitochondrial MnSOD was not associated with the ER enriched fraction. Catalase gave a very weak signal in the ER enriched fraction. No topoisomerase I was detected in the ER enriched fraction. The ascorbate transporter SVCT1 gave a weak signal in the ER enriched fraction. The strongest signal for SVCT1 was found in the supernatant fraction that contained surface cellular membranes. We measured the intensities of each protein in the different fractions and performed a principal component analysis (PCA). The PCA indicated that the fractionation was consistent from one animal to another and genotype independent.

Three mice of each cohort constituting biological triplicates were used for label-free quantification analyses. Using stringent criteria (1% FDR based on a target/decoy database search), we could reliably identify a total of 28,366 unique peptides. Label-Free Quantification (LFQ) signal intensity for each peptide in the different ER enriched samples is indicated in the S1 Table (Note that 182 peptides were considered contaminants based on the Andromeda software and are shown in gray in the list of S1 Table). Overall, the identified peptides mapped to 4058 proteins across all samples. However, only 3097 proteins were quantifiable with abundances above the lower limit of quantification when we compared WT and *Wrn*^*Δhel/Δhel*^ liver samples. In other words, 961 proteins out of the 4058 mapped proteins were not identified or quantifiable when we compared WT and *Wrn*^*Δhel/Δhel*^ liver samples. Similarly, only 3117 proteins were quantifiable (with abundances above the lower limit of quantification) when we compared WT and vitamin C treated *Wrn*^*Δhel/Δhel*^ liver samples. Finally, only 3127 proteins were quantifiable (with abundances above the lower limit of quantification) when we compared untreated *Wrn*^*Δhel/Δhel*^ and vitamin C treated *Wrn*^*Δhel/Δhel*^ liver samples.

**Fig 3:**
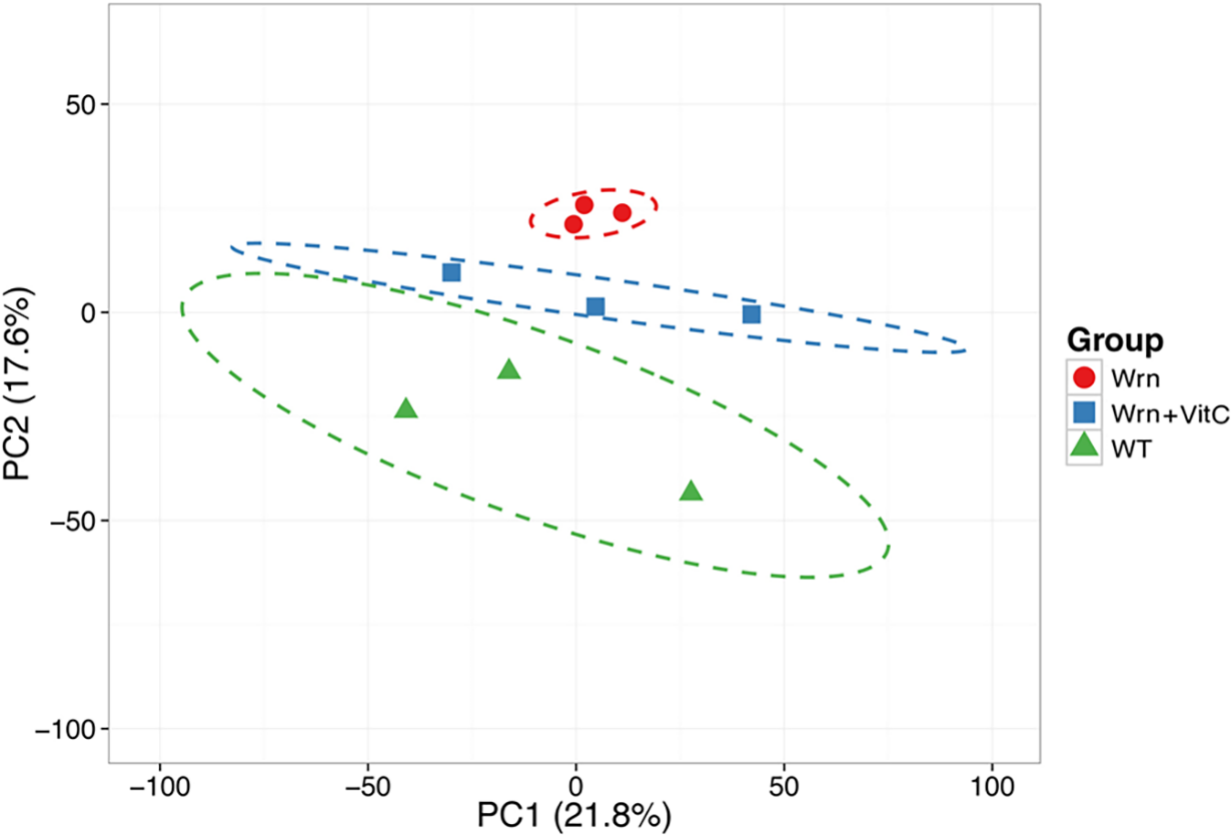
Principal component analysis (PCA) graph demonstrating the effect of vitamin C on the ER proteomic profiles of *Wrn*^*Δhel/Δhel*^ mice compared to wild type mice. The three wild type mice are represented by green triangles, the three *Wrn*^*Δhel/Δhel*^ mice are represented by red circles, and the three vitamin C treated *Wrn*^*Δhel/Δhel*^ mice are represented by blue squares. X and Y axis show principal component 1 and principal component 2 that explain 21.8% and 17.6% of the total variance, respectively. Prediction ellipses are such that a new observation from the same group will fall inside the ellipse with a probability of 0.95.

Using the LFQ data, we explored the proteomic profile of each mouse cohort by employing a principal component analysis. In this analysis, we only kept the 3074 proteins that were considered quantifiable (i.e. if at least two replicate values were quantifiable within a mouse cohort) in all the three cohorts. The PCA approach showed a good clustering of the three *Wrn*^*Δhel/Δhel*^ mice (Fig 3). Wild type animals showed more variation between individual biological triplicates but did not overlap with the *Wrn*^*Δhel/Δhel*^ mutant mice. The three vitamin C treated *Wrn*^*Δhel/Δhel*^ mice were clustered together between the untreated wild type and *Wrn*^*Δhel/Δhel*^ mice. Thus, although vitamin C treatment pushed the overall proteomic profile of *Wrn*^*Δhel/Δhel*^ mice toward the wild type profile, it did not overlap with it.

**Fig 4:**
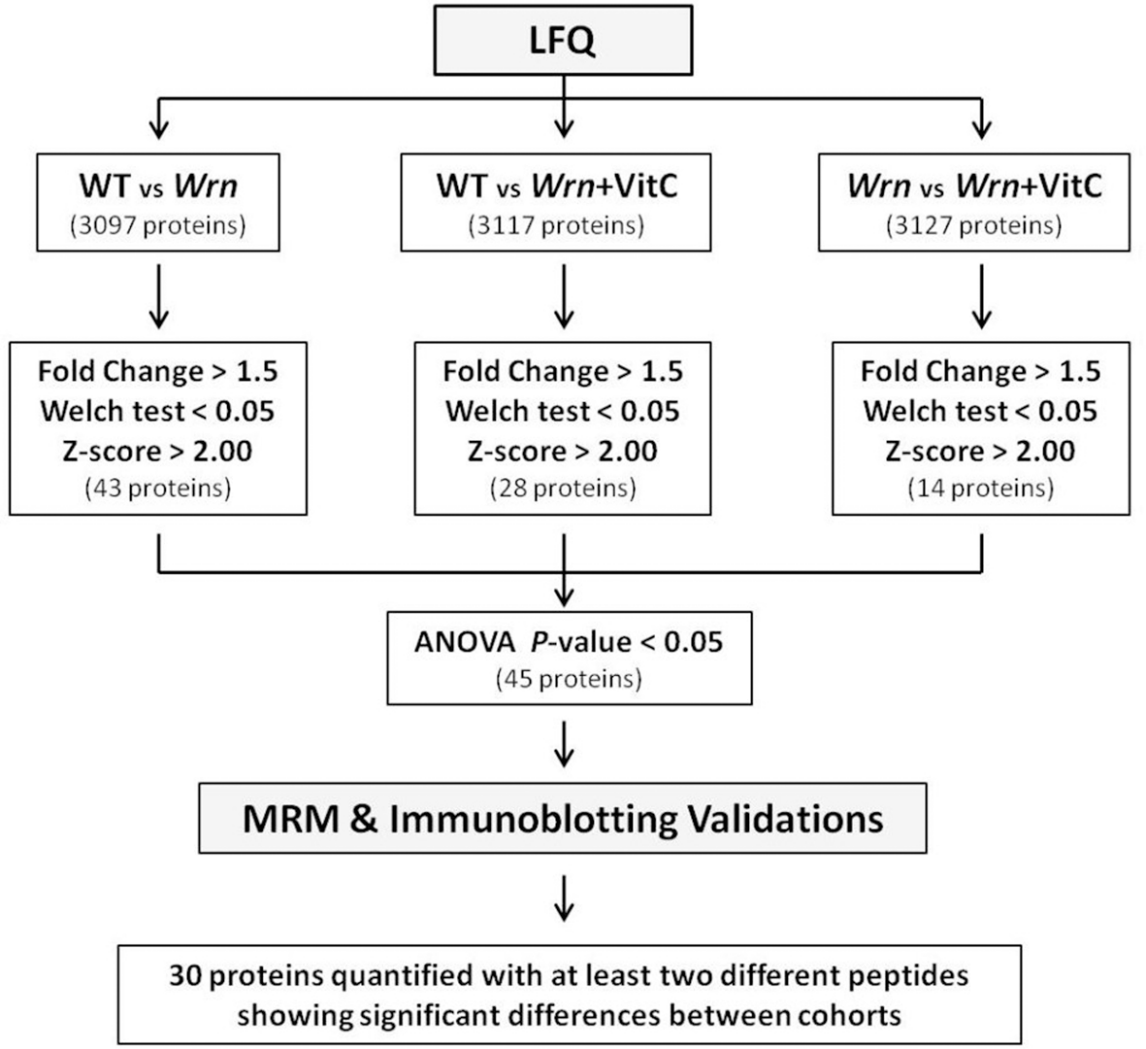
Schematic representation of the different steps undertaken to obtain a list of protein differentially expressed between our different mouse cohorts at a statistical significant level. The number of proteins identified in each step is indicated in parentheses. WT = wild type mice; Wrn = *Wrn*^*Δhel/Δhel*^ mice; Wrn+VitC = *Wrn*^*Δhel/Δhel*^ mice treated with 0.4% vitamin C (w/v) in drinking water since weaning.

To generate a list of proteins significantly altered between the different cohorts of mice, we first identified proteins with at least a 1.5-fold difference between two groups of mice. We then considered these differences to be significant with a Welch’s *t*-test *P*-value < 0.05 and an absolute Z-score > 2.0. When we compared WT versus *Wrn*^*Δhel/Δhel*^ mice, 43 proteins passed these criteria. When we applied the same criteria for WT versus *Wrn*^*Δhel/Δhel*^ treated with vitamin C and untreated *Wrn*^*Δhel/Δhel*^ versus *Wrn*^*Δhel/Δhel*^ treated with vitamin C comparisons, we obtained 28 and 14 proteins, respectively. The list of these proteins is highlighted in yellow in the corresponding comparison tabs of S1 Table. Since we were comparing three groups of mice, we finally applied a one-way ANOVA to each protein that were identified with a Welch’s *t*-test *P*-value < 0.05 and an absolute Z-score > 2.0 in the different comparisons shown in the S1 Table. A schematic of all the statistical analyses performed to identify protein expression significantly altered between all groups of mice is presented in Fig 4. A total of 45 proteins were significantly altered with an ANOVA *P*-value < 0.05 in either group of mice (S2 Table).

### Proteins selected for MRM and immunoblottings in the validation phase of the LFQ

Twenty-five proteins were selected for the validation phase by the MRM method in an independent set of WT, *Wrn*^*Δhel/Δhel*^, and vitamin C treated *Wrn*^*Δhel/Δhel*^ mice (three new mice in each group). The peptides that were chosen for the MRM analyses are shown in S3 Table. The raw quantification results from the MRM analyses for these peptides are given in the S4 Table. Eleven of these proteins showed an ANOVA *P*-value < 0.05 based on the LFQ data (S5 Table). We noticed a correlation between the LFQ data and the MRM results when the LFQ showed an ANOVA *P*-value < 0.05 with more than two peptides quantifiable by LFQ (S5 Table). Tmem254b and Tpd52 proteins showed similar differential expression between WT versus *Wrn*^*Δhel/Δhel*^ mice, WT versus vitamin C treated *Wrn*^*Δhel/Δhel*^ mice, and untreated versus vitamin C treated *Wrn*^*Δhel/Δhel*^ mice when we compared the LFQ and the MRM data (S5 Table). There was also a correlation between the differential expressions of Pigr and Tfrc between the different groups of mice when we compared the LFQ and the MRM results. However, the differential expression was much lower by MRM (S5 Table). Cyp2b9, Igha, and Selenbp2 showed a good correlation for the LFQ and MRM data between WT versus *Wrn*^*Δhel/Δhel*^ mice and WT versus vitamin C treated *Wrn*^*Δhel/Δhel*^ mice (S5 Table). The expression alterations between untreated and vitamin C treated *Wrn*^*Δhel/Δhel*^ mice were below 1.5-fold for both the LFQ and MRM data of Cyp2b9, Igha, and Selenbp2 proteins and were thus not considered different. Although the calculated Welch’s test *P*-values for most comparisons with the MRM quantifications were not significant (*P* > 0.05), there was a good tendency (*P* < 0.2) for most comparisons between groups of mice.

Nucb2 was identified by only one peptide in the LFQ. As this peptide could not be measured by the MRM method, a different peptide was used for the MRM. Interestingly, this second peptide confirmed the differential expression between groups of mice that we originally found by the LFQ method. More precisely, Nucb2 protein levels were lower in untreated and vitamin C treated *Wrn*^*Δhel/Δhel*^ mice compared to untreated WT mice (*P*-values = 0.07 and 0.06, respectively). The difference between untreated and vitamin C treated *Wrn*^*Δhel/Δhel*^ mice were less than 1.5-fold and not significant (*P*-value = 0.53) (S5 Table). A similar strategy was used for Gstm7 and Synpo2 but the MRM results showed opposite protein expression when compared to the LFQ data. Finally, the MRM results showed less than 1.5-fold difference in protein expression (or in opposite direction) when the ANOVA from the LFQ method was not significant (S5 Table).

**Fig 5:**
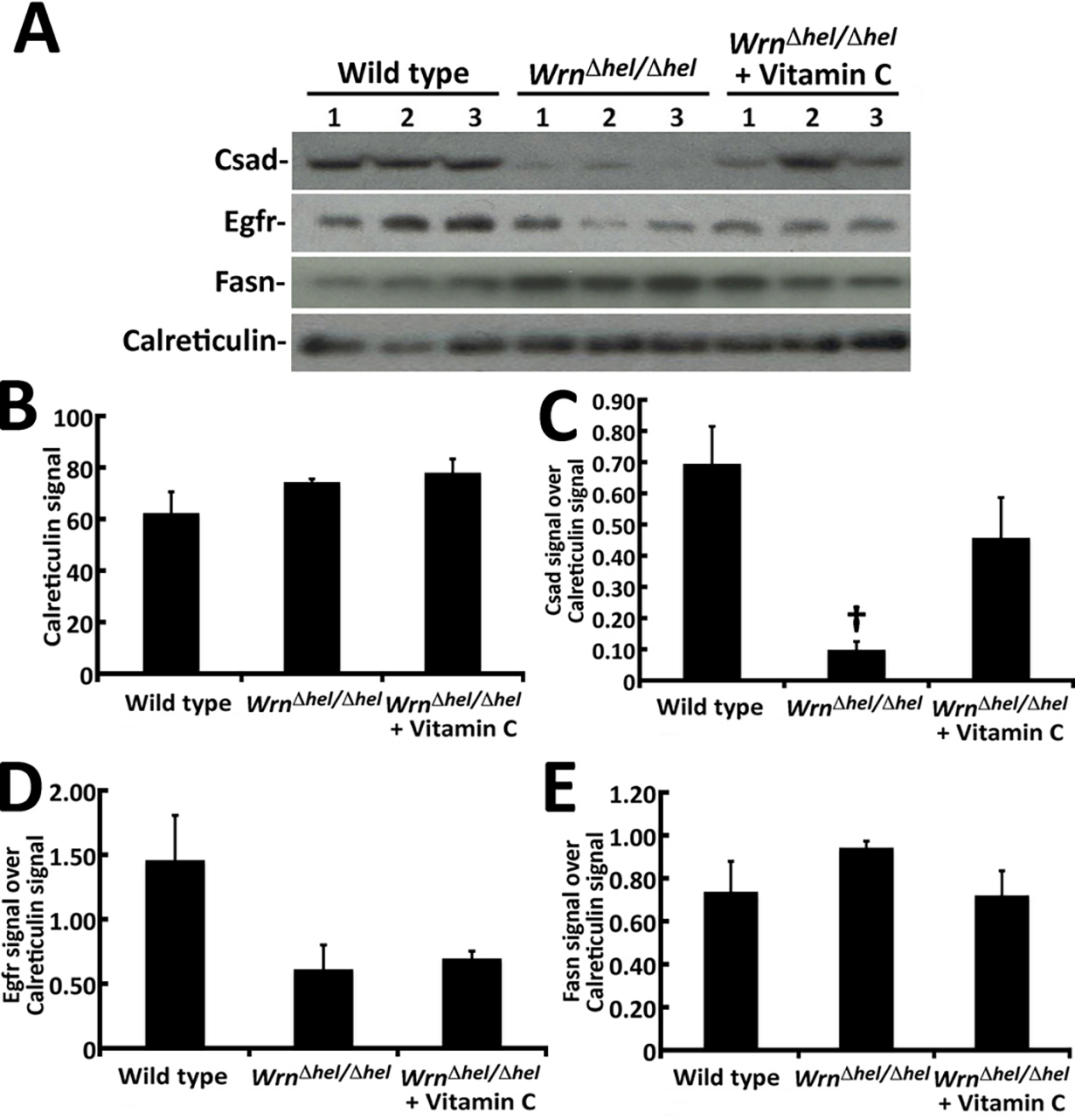
Validation of protein levels of various gene products in our different mouse cohorts by immunoblotting. (A) Example of western blots showing protein levels of Csad, Egfr, Fasn, and calreticulin in our mouse cohorts. (B) Signal intensity of ER marker calreticulin. (C) Ratio of Csad over calreticulin signal from the western blots. (Tukey post ANOVA test: †*P* < 0.05 compared to wild type mice). (D) Ratio of Egfr signal over calreticulin signal from the western blots. (ANOVA *P*-value = 0.07). (E) Ratio of Fasn signal over calreticulin signal from the western blots (ANOVA *P*-value = 0.33).

Four proteins were also selected for further qualification of the LFQ data by immunoblotting on the same samples that were used for the MRM method. They included Csad, Egfr, Fasn, and the loading control for western blot calreticulin (Calr). Based on the LFQ data, Calr did not show a significant difference between the three groups of mice (ANOVA *P*-values > 0.05; S5 Table). The immunoblot analyses confirmed these results (Fig 5A and 5B).

Based on the LFQ data, Csad was decreased in *Wrn*^*Δhel/Δhel*^ mice treated with or without vitamin C. The immunoblot analysis indicated a significant decrease of Csad in *Wrn*^*Δhel/Δhel*^ mice and a tendency to decrease in vitamin C treated *Wrn*^*Δhel/Δhel*^ mice compared to WT mice (Fig 5C). Interestingly, all the ratios from the Csad immunoblot data were exactly in the same directions as the ratios of the MRM data (compare both tabs of S5 Table).

The immunoblot analyses indicated that Egfr was decreased by more than two-fold in *Wrn*^*Δhel/Δhel*^ mice treated with or without vitamin C compared to WT mice (Fig 5D and S5 Table). Based on the LFQ data, Egfr levels were decreased in *Wrn*^*Δhel/Δhel*^ mice treated with or without vitamin C by more than 1.6-fold (S5 Table) compared to WT mice. The difference between untreated and vitamin C treated *Wrn*^*Δhel/Δhel*^ mice was below 1.5-fold.

Finally, although the ANOVA *P*-value for Fasn in the LFQ was < 0.05 (S5 Table), the calculated Z-scores for the different comparisons were < 2.0 (S1 Table) suggesting that Fasn levels in our cohorts may not be significantly different using another quantification technique. Our immunoblot analyses on Fasn confirmed this suggestion (Fig 5A and 5E and S5 Table).

Overall, these results indicated that the MRM and the immunoblot analyses validated the differential expression of various proteins between our different mouse cohorts for proteins exhibiting an ANOVA *P*-value < 0.05 and an absolute Z-score > 2.0 in the different group comparisons. In addition, two different peptides for the same protein had to show an identical differential expression. These criteria were used to generate a final list of differentially expressed ER proteins in our different mouse cohorts.

### Classification of the proteins exhibiting significant alterations between our cohorts of mice

**Fig 6:**
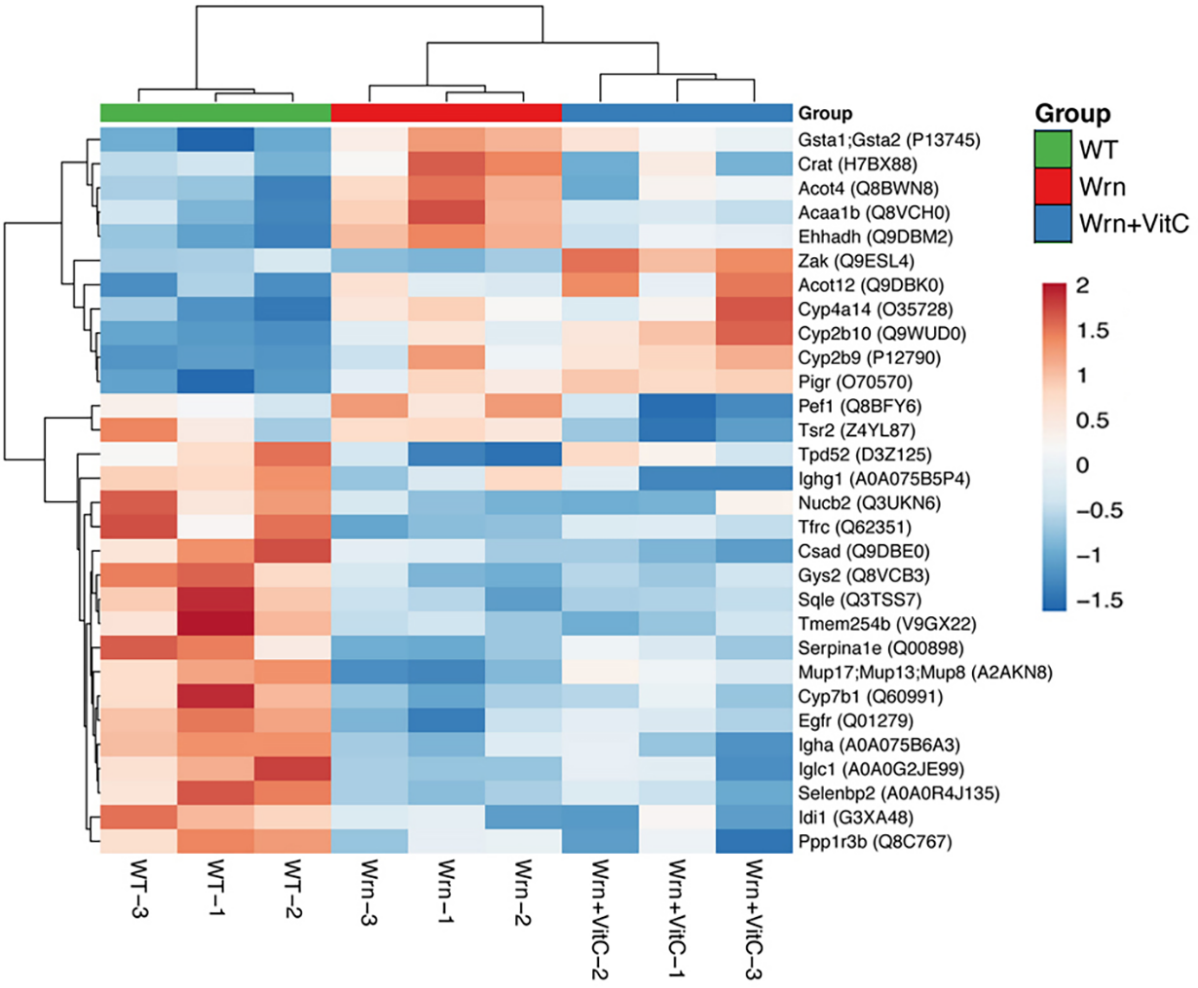
List of proteins showing significant differential expression in our different mouse cohorts (or groups). Heatmap depicting the Z-score of log base ten of normalized intensities from the LFQ data for each protein (rows) between individual (columns) wild type and *Wrn*^*Δhel/Δhel*^ mice treated with or without vitamin C. Columns and rows are reordered by hierarchical clustering using the genotype and vitamin C treatments. The protein names are indicated on the right with their protein ID numbers in parentheses.

From the information obtained with the MRM and immunoblot analyses, we generated a list of 30 proteins identified by at least two different peptides (in the LFQ and/or MRM) showing significant expression differences between WT, *Wrn*^*Δhel/Δhel*^, and *Wrn*^*Δhel/Δhel*^ mice treated with vitamin C. A summary of the 30 proteins significantly altered in at least one of the groups is given in a form of a heatmap in Fig 6. The dendrogram on the left hand side of the heatmap indicates the proteins that clustered together based on their expression profile in each group of mice. A heatmap with the means of protein intensities for each individual mouse cohort (or group of three replicates) is shown in the supplementary S1 Fig. Interestingly, protein levels of Crat, Acot4, Acaa1b, and Ehhadh were increased in *Wrn*^*Δhel/Δhel*^ mice compared to wild type mice. Vitamin C treatment decreased these proteins near wild type levels. In contrast, Tpd52 protein levels were decreased in *Wrn*^*Δhel/Δhel*^ mice compared to wild type mice but increased upon vitamin C treatment.

The PANTHER classification system was used to assign differentially expressed proteins by their functions in specific biological processes and cellular compartments. The proteins in the heatmap were part of cellular microbodies, peroxisomes, the endoplasmic reticulum, the microsome and vesicular fractions as expected from the ER enrichment methods that we performed on the liver of our mice (Table 1). Categorization of all the proteins from the heatmap revealed that the fatty acid and steroid metabolism pathways are differently regulated in these different cohorts of mice (with a Bonferroni *P*-value = 0.0137).

**Table 1:** Ontology analyses of differentially expressed proteins in our mouse cohorts.

**Cellular compartments**	**Bonferroni *P*-value**	**Proteins**
Cytoplasmic part	0.0002	Egfr, Cyp2b9, Ehhadh, Selenbp2, Crat, Tpd52, Cyp2b10, Acot4, Cyp7b1, Tfrc, Sqle, Nucb2, Acot12, Gys2, Cyp4a14, Idi1, Acaa1b
Microbody	0.0007	Ehhadh, Crat, Idi1, Acaa1b, Acot4
Peroxisome	0.0007	Ehhadh, Crat, Idi1, Acaa1b, Acot4
Endoplasmic Reticulum	0.0052	Cyp7b1, Sqle, Cyp2b9, Nucb2, Crat, Cyp2b10, Tpd52, Cyp4a14
Microsome	0.0057	Cyp7b1, Sqle, Cyp2b9, Cyp2b10, Cyp4a14
**Biological pathways**	**Bonferroni *P*-value**	**Proteins**
Fatty acid and steroid metabolism	0.0137	Cyp7b1, Cyp2b9, Ehhadh, Acot12, Crat, Cyp2b10, Cyp4a14

### Identification of the ER stress proteins by the LFQ technique

**Fig 7:**
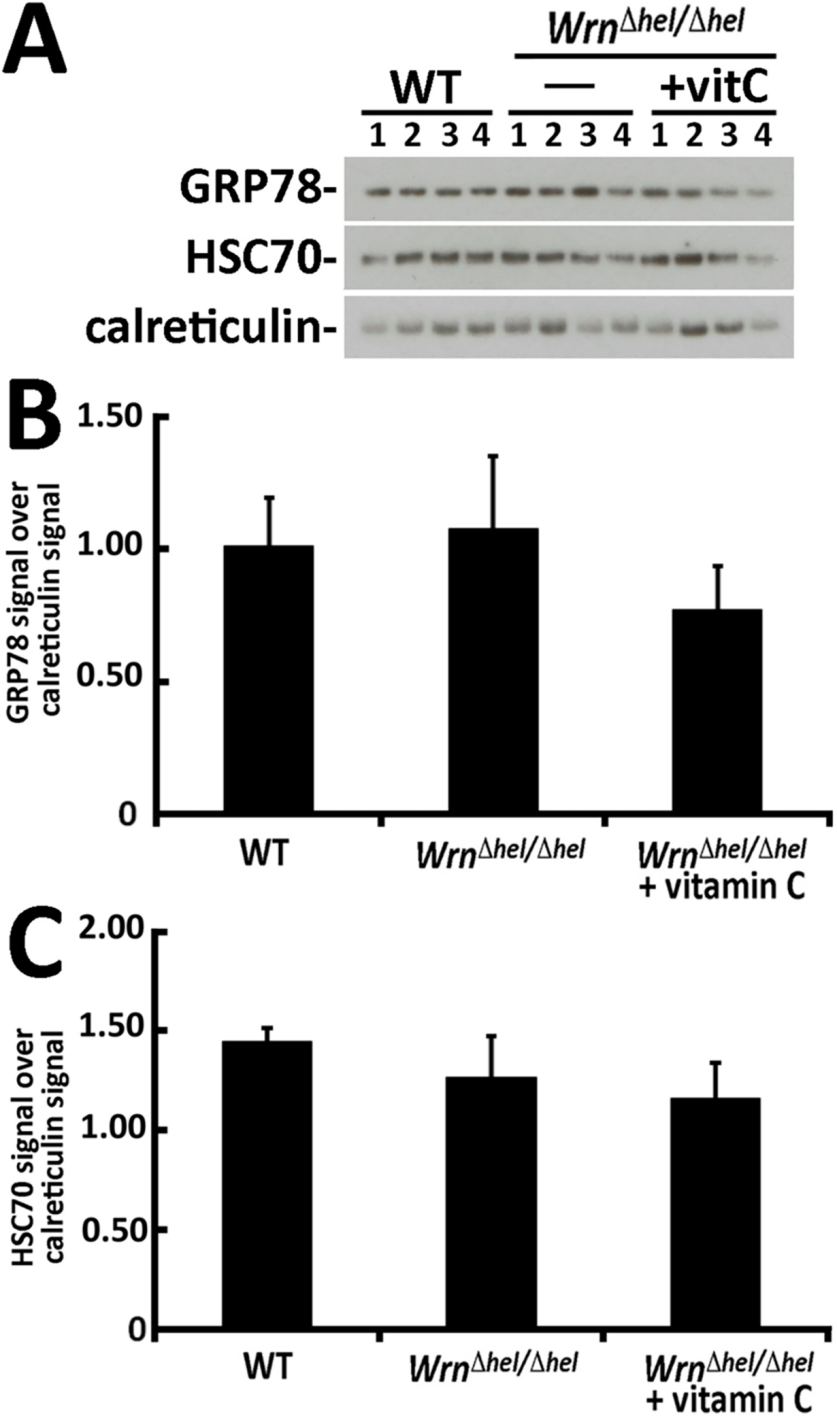
Protein levels of GRP78 and HSC70 proteins in the ER enriched fractions from mice. (A) Example of western blots showing protein levels of GRP78, HSC70, and calreticulin in liver ER enriched fractions of mice. (B) Ratio of GRP78 signal over calreticulin signal from the western blots. (C) Ratio of HSC70 signal over calreticulin signal from the western blots. (All Tukey post ANOVA and *t*-tests *P*-values > 0.05). Bars in all histograms represent SEM of four mice.

We determined whether the ER stress proteins analyzed in Fig 1 were identified by our mass spectrometry analyses (S1 Table). PERK and IRE1α were not identified by the LFQ technique. The eIF2α protein was identified by the LFQ as Eif2a peptides and showed no significant difference between our three mouse cohorts (S1 Table). Accordingly, the immunoblot analysis of total Eif2a protein showed no difference between our three mouse cohorts (Fig 1A). The name of the gene encoding GRP78 is Hspa5 and was identified by mass spectrometry in liver ER enriched fractions. Our LFQ analysis did not show a significant difference between our mouse cohorts (S1 Table). The gene name coding for HSC70 is Hspa8 and was also identified by mass spectrometry in liver ER enriched fractions. Our LFQ analysis did not show a significant difference between our mouse cohorts (S1 Table) confirming the immunoblot results in Fig 1. Since the immunoblots of Fig 1 were performed on total liver lysates, we re-examined the levels of GRP78 (Hspa5) and HSC70 (Hspa8) directly in liver ER enriched protein fractions. As indicated in Fig 7, the immunoblot results indicated no significant difference (*P*-values > 0.05) between the levels of GRP78 and HSC70 in the ER enriched fractions of our different mouse cohorts in agreement with the LFQ results.

### Comparison of the proteome profile to the mRNA profile of wild type and *Wrn*^*Δhel/Δhel*^ liver tissues

In a previous study, we compared the liver transcriptome profiles of three-month old *Wrn*^*Δhel/Δhel*^ mice to age-matched wild type animals [16]. We thus compared the list of altered mRNAs in our *Wrn*^*Δhel/Δhel*^ mice (compared to wild type animals) to the list of proteins identified by mass spectrometry in the ER enriched fractions of *Wrn*^*Δhel/Δhel*^ and wild type mice (from the S1 Table). Out of the 328 RNAs that were identified as significantly different (by at least 1.5-fold) between *Wrn*^*Δhel/Δhel*^ and wild type mice, 89 mRNAs encoded the proteins identified by our LFQ analysis in our ER enriched fractions from the liver of *Wrn*^*Δhel/Δhel*^ and wild type mice (S6 Table). However, only seven genes showed a significant 1.5-fold difference or more between wild type and *Wrn*^*Δhel/Δhel*^ mice at both the mRNA and protein levels (Table 2). Importantly, the differential expression of Nucb2, Tfrc, and Egfr were validated by MRM or immunoblotting methods. In contrast, the alteration of Oat protein levels between WT and *Wrn*^*Δhel/Δhel*^ mice was not confirmed by the MRM method (S5 Table). Lastly, although the MRM technique showed an increase of Acaab1 protein level in *Wrn*^*Δhel/Δhel*^ mice compared to WT animals, the increase was less than 1.5-fold. These results indicate that the levels of 84 identified ER proteins (94%) in ER enriched fractions were not reflected by the levels of their corresponding mRNA for the *Wrn*^*Δhel/Δhel*^ versus wild type mice comparison in our analyses.

**Table 2:** List of proteins correlating with mRNA expression alteration in *Wrn*^*Δhel/Δhel*^ mice versus wild type animals*.

Gene name	protein fold change	mRNA fold change
Nucb2	-3.44	-4.66
Sqle	-2.90	-2.55
Tfrc	-2.45	-2.01
Egfr	-2.18	-1.81
Acaa1b	2.19	2.26
Gsta2	2.95	2.27
Oat	3.01^a^	2.38

*For proteins with a Welch’s *t*-test *P*-value < 0.05 and an absolute Z-score > 2.0 (from the LFQ of S1 Table).

^a^Not confirmed by the MRM method.

We also compared the list of altered mRNAs in our vitamin C treated *Wrn*^*Δhel/Δhel*^ mice (compared to wild type animals) [16] to our list of ER proteins identified by mass spectrometry (S1 Table). Out of the 186 mRNAs that were identified as significantly different (by at least 1.5-fold), 56 mRNAs encoded proteins identified in our ER enriched fractions from the liver of vitamin C treated *Wrn*^*Δhel/Δhel*^ and untreated wild type mice (S7 Table). However, only four genes showed a 1.5-fold difference or more between wild type and vitamin C treated *Wrn*^*Δhel/Δhel*^ mice at both the mRNA and protein levels (Table 3). Note that the peptides corresponding to Gsta1 and Gsta2 identified during the LFQ method could not differentiate these two proteins unlike the mRNA probes (S7 Table). Thus, the increased levels of 52 identified ER proteins (93%) in ER enriched fractions were not reflected by the levels of their corresponding mRNA levels in the vitamin C treated *Wrn*^*Δhel/Δhel*^ mice versus wild type mice comparison.

**Table 3:** List of proteins correlating with mRNA expression alteration in *Wrn*^*Δhel/Δhel*^ mice treated with vitamin C versus untreated wild type animals*.

Gene name	protein fold change	mRNA fold change
Cyp2b10	2.77	1.69
Gsta1	2.35^a^	1.86
Gsta2	2.35^a^	2.93
Cyp2b9	12.90	3.03

*For proteins with a Welch’s *t*-test *P*-value < 0.05 and an absolute Z-score > 2.0 (from the LFQ of S1 Table).

^a^The peptides identified in the LFQ could not differentiate between Gsta1 and Gsta2 proteins.

## Discussion

We have previously observed an increased oxidative stress in the ER enriched fraction of liver from *Wrn*^*Δhel/Δhel*^ mice compared to wild type animals [16]. In the present study, we found an alteration in the level and activation of different ER stress response markers in *Wrn*^*Δhel/Δhel*^ mice compared to wild type animals (Fig 1). We further explored this phenotype by examining the proteomic profiles of the ER enriched fractions of *Wrn*^*Δhel/Δhel*^ mice treated with or without vitamin C in drinking water. A significant enrichment of ER component proteins was identified along some peroxisomal and microsomal associated proteins using a Label-Free Quantification (LFQ) mass spectrometry strategy [20]. Since peroxisomal and microsomal bodies can originate from an ER membrane template, it is not surprising to detect proteins associated with these organelles in our ER enriched fractions [27]. Accordingly, we detected by western analyses the peroxisomal protein catalase in our ER enriched fraction, although the signal was weak (Fig 2B). Approximately 3074 proteins were identified and quantified (with abundances above the lower limit of quantification) in all our three mouse cohorts (wild type, *Wrn*^*Δhel/Δhel*^, and vitamin C treated *Wrn*^*Δhel/Δhel*^ mice). A principal component analysis of the LFQ data indicated that the overall proteomic profiles of wild type and *Wrn*^*Δhel/Δhel*^ mice were different and not overlapping. Although vitamin C treatment pushed the overall proteomic profile of *Wrn*^*Δhel/Δhel*^ mice toward the wild type profile, it did not overlap with it (Fig 3). These results indicate that the ER enriched fractions exhibit different proteomic profile in our three mouse cohorts. Proteomic profiling of lysosomal, mitochondrial, nuclear, cytosolic, and membrane fractions will be required to have a more complete picture of the impact of vitamin C on the liver tissue of *Wrn*^*Δhel/Δhel*^ mice.

We used the Multiple Reaction Monitoring (MRM) and immunoblotting methods in the qualification phase of our LFQ studies to generate a list of proteins up or down-regulated in our different mouse cohorts (Fig 6). More specifically, the MRM and the immnoblotting experiments were used to define stringent statistical criteria to obtain a list of significantly altered proteins in the ER enriched fractions between our different mouse cohorts from the LFQ data (Fig 4). It is important to mention that we used different mice within each cohort for the LFQ and the MRM experiments. Although this strategy certainly introduced a certain degree of variability in our experiments due to the different processing times during the ER fractionation procedures, the fact that we were able to confirm differential expression of several proteins by two different methods on different sets of mice increased our confidence in the LFQ data mining phase. One issue that we faced during the MRM validation phase is that several peptides identified by the LFQ method could not be identified or quantified by the MRM method in our samples as each method requires different HPLC and mass spectrometry equipments involving different selectivity and sensitivity. Additionally, in order to provide an accurate and reproducible quantification, the peptides with variable stability were avoided. This includes peptides containing residues susceptible to artifactual modifications during sample preparation such as methionine and peptides containing sequences that commonly result in missed cleavages [23]. There are two possible outcomes from such an issue. In one hand, it is possible that using different peptides in the MRM may represent different spliced variant of a protein that could be a potential source of false-negative results in our validation experiments. In addition, some peptides identified in the LFQ could not differentiate between proteins of the same family of enzymes. For example, we could not differentiate between the levels of Gsta1 and Gsta2 proteins in our samples and there is no alternative peptide that can be used in the MRM method to confidently quantify either enzyme of the same family. On the other hand, two different peptides quantified by two different methods may give the same result enforcing our confidence in our findings. The decreased level of Nucb2 proteins in *Wrn*^*Δhel/Δhel*^ mice compared to wild type animals is one good example. The peptide used for the MRM was different from the one identified by LFQ but showed the same decreased level of Nucb2 proteins in *Wrn*^*Δhel/Δhel*^ mice (S5 Table). Another issue that we also faced during the MRM validation phase is the Welch’s statistical *P*-values that were above the usual 0.05 cut-off values for significance between our different groups of mice. We noticed large inter individual differences during the relative MRM quantifications of several peptides within each group of mice. To get a better picture of protein expression between groups of mice, we will in the future obtain more than three biological replicates per mouse cohorts. Despite the limitations of the MRM method, we were able to validate several proteins differentially expressed (with descent trends as indicated by *P*-values < 0.1) in our different mouse cohorts allowing us to focus and understand the potential functions of these proteins in WS pathology in future studies.

Categorization of the proteins exhibiting significantly altered levels between our different mouse cohorts using the PANTHER bioinformatics tool revealed a significant change in the biological process affecting fatty acid and steroid metabolism pathways (Table 1). Alterations in protein levels involved in fatty acid metabolism is consistent with previous metabolomics data showing altered lipid synthesis in the liver and anomalies in the serum ratio of very long chain to short chain lysophosphatidylcholines in the *Wrn*^*Δhel/Δhel*^ mice [13–16]. Interestingly, vitamin C treatment of *Wrn*^*Δhel/Δhel*^ mice tended to re-establish the levels of some proteins near wild type levels in the ER enriched fractions (Fig 6). Such proteins included Crat, Acot4, Acaab1, Ehhadh, and Tpd52. The carnitine O-acetyltransferase (Crat) enzyme catalyzes the reversible transfer of acyl groups from an acyl-CoA thioester to carnitine and regulates the ratio of acyl-CoA/CoA. It is thus a key metabolic pathway enzyme that plays an important role in energy homeostasis and fat metabolism. Crat is found in both the mitochondria and the peroxisome [28]. The acyl-CoA thioestherase-4 enzyme (Acot4) is responsible for the termination of beta-oxidation of dicarboxylic acids of medium-chain length with the concomitant release of the corresponding free acids [29]. The 3-ketoacyl-CoA thiolase B protein (Acaab1) is a peroxisomal enzyme that catalyzes the thiolytic cleavage of straight chain 3-ketoacyl-CoAs. Acaab1 deficient mice have been shown to exhibit disturbances in cholesterol, bile acid, and glucose metabolism [30]. The enoyl-CoA hydratase and 3-hydroxyacyl CoA dehydrogenase enzyme (Ehhadh) has two catalytic activities and is involved in the peroxisomal beta-oxidation pathway [31]. Finally, the tumor protein D52 (Tpd52) is a potential suppressor of hepatocellular carcinoma [32]. It is detected in the lipid droplet fraction and promotes intracellular lipid storage [33]. Thus, the current results substantiate the metabolomic evidence that have established a dysregulation of peroxisomal lipid metabolism in *Wrn*^*Δhel/Δhel*^ mice, which can be improved by vitamin C treatment [13–16]. Importantly, vitamin C is a cofactor in the biosynthesis of carnitine, a molecule required for the oxidation of fatty acids. A reduction in the ability to oxidize fat will contribute to dyslipidemia [34, 35]. Vitamin C is also a cofactor for the enzyme 7α-hydroxylase catalyzing the conversion of cholesterol to bile acids. Low levels of vitamin C results in reduced whole body excretion of cholesterol in animals [34]. Accordingly, we previously found an increase of serum cholesterol in *Wrn*^*Δhel/Δhel*^ mice [13]. Since cholesterol is important in steroidogenesis, an alteration of cholesterol levels *in vivo* will impact steroid metabolism [34].

In addition to a defect in the quality of DNA repair, the lack of a functional WRN protein is believed to affect transcription as well [8, 36–38]. Transcriptomic profiling analyses of the liver from *Wrn*^*Δhel/Δhel*^ and wild type mice have suggested a similar conclusion [14, 16]. However, no study at the protein level has confirmed this hypothesis in mouse. In the current study, we found major discrepancies between microsomal, peroxisomal, or ER specific encoding mRNA levels and their corresponding protein levels (measured by LFQ) in our different mouse cohorts. When we compared *Wrn*^*Δhel/Δhel*^ and wild type mice, the levels of only seven proteins reflected the levels of their corresponding mRNAs (Table 2). When we compared the vitamin C treated *Wrn*^*Δhel/Δhel*^ mice and wild type mice, the levels of four proteins reflected their mRNA levels (although the LFQ method could not differentiate between Gsta1 and Gsta2 proteins). In our different mouse group comparisons, more than 93% of the differentially expressed mRNAs, for which a protein was identified and quantified by LFQ, did not lead to a significant difference at the protein level. There are several potential reasons for these discrepancies. The proteomic studies were performed on the liver of five-month old mice, while the transcriptomic data was obtained from the liver of three-month old mice. It is unknown to what extent a two-month difference in age can affect the mRNA and protein levels in the liver of mice. Nonetheless, analyses of the transcriptomic data indicated significant changes in mRNAs encoding enzymes involved in lipid metabolism and associated with the ER even in the liver of three-month old *Wrn*^*Δhel/Δhel*^ mice [16]. Although we focused on the ER enriched fraction of mouse liver tissue, a limitation of the LFQ is the complexity of the protein sample under analysis, which can complicate the mass spectra display and relative quantification of individual peptides between samples. As such, the mass signals of low intensity (within the background noise) or of high intensity (detector saturation) may not allow a correct identification [20]. If we had used total lysates from our liver samples, the complexity of the samples would have been much greater leading to a higher false discovery rate. Since the mRNA profiles will in theory reflect total protein levels in a tissue, the ER fractionation procedure that we used in this study may have provided only a fraction of the total proteins encoded by these mRNAs present in a liver sample. This could have been partly responsible for the discrepancies observed between the transcriptomic and the proteomic profiles from the liver tissues of our mouse cohorts.

Independently of the technical limitations associated with the transcriptomic or proteomic strategies, our data is consistent with previous studies indicating that the correlation between mRNA and protein levels are far from perfect in several biological systems [20, 39, 40]. Furthermore, changes at the transcriptional level may not lead to similar changes at the translational level for specific gene product. It is possible that the difference in ER protein levels between groups of mice may be mainly due to post-transcriptional regulation of the gene products. Accordingly, we found an increased phosphorylation of the translation initiation factor eIF2α in *Wrn*^*Δhel/Δhel*^ mice compared to wild type animals (Fig 1). This phosphorylation was reversed upon vitamin C treatment suggesting that the increased oxidative stress observed in the ER enriched fraction of *Wrn*^*Δhel/Δhel*^ mice [16] is likely the culprit for this change in our mice. Phosphorylation of eIF2α results in the formation of a stalled 43S ternary complex that causes a general decrease in translation of most proteins. However, some selected proteins with internal ribosomal entry sites, are translated more efficiently and hence their protein levels actually increase [41, 42]. Future studies should focus on the translational aspect of mRNAs to learn more about the function of differentially expressed proteins in the pathology of WS.

## Conclusion

To conclude, the discovery and qualification phase proteomic studies that we have undertaken in the present work indicate changes in the levels of several microsomal, peroxisomal, and ER associated proteins involved in fatty acid metabolism in the liver of *Wrn*^*Δhel/Δhel*^ mice. These results are consistent with previous metabolomic results [16]. Although we found discrepancies between the mRNA levels and the corresponding amounts of the protein encoded by these mRNAs, our findings provide clues to the ER responses upon oxidative stress in *Wrn*^*Δhel/Δhel*^ mice that ultimately lead to the potential differential translation of several mRNAs. Our data also provide information on proteins that likely affect the course of the pathology observed in our WS mouse model.

## Supporting information

S1 Fig List of proteins showing significant differential expression in our different mouse cohorts (or groups). Heatmap depicting the Z-score of log base ten of the means normalized intensities of each protein (rows) between groups of mice (columns).
(TIF)

S1 Table Raw data of the peptides and proteins identified and quantified by the Label-Free Quantification method. (XLSX)

S2 Table Label-Free Quantification of proteins between wild type and Wrn mutant mice treated with or without vitamin C exhibiting significant difference of expression based on ANOVA. (XLSX)

S3 Table Peptides used for the Multiple Reaction Monitoring analyses. (XLSX)

S4 Table Raw data from the Multiple Reaction Monitoring analyses used for the relative quantifications of proteins. (XLSX)

S5 Table Quantification of selected proteins by Multiple Reaction Monitoring and immunoblotting in the validation phase of the study. (XLSX)

S6 Table List of genes found to be differentially expressed at the mRNA and protein levels in the liver of Wrn mutant compared to the wild type mice. (XLSX)

S7 Table List of genes found to be differentially expressed at the mRNA and protein levels in the liver of vitamin C treated Wrn mutant compared to the wild type mice. (XLSX)
