# Supplementary figures and images for: Vitamin C alters the amount of specific endoplasmic reticulum associated proteins involved in lipid metabolism in the liver of mice synthesizing a nonfunctional Werner syndrome (Wrn) mutant protein
Source: PLoS One. 2018 Mar 1;13(3):e0193170. doi: 10.1371/journal.pone.0193170 (PMC5832228; doi:10.1371/journal.pone.0193170)

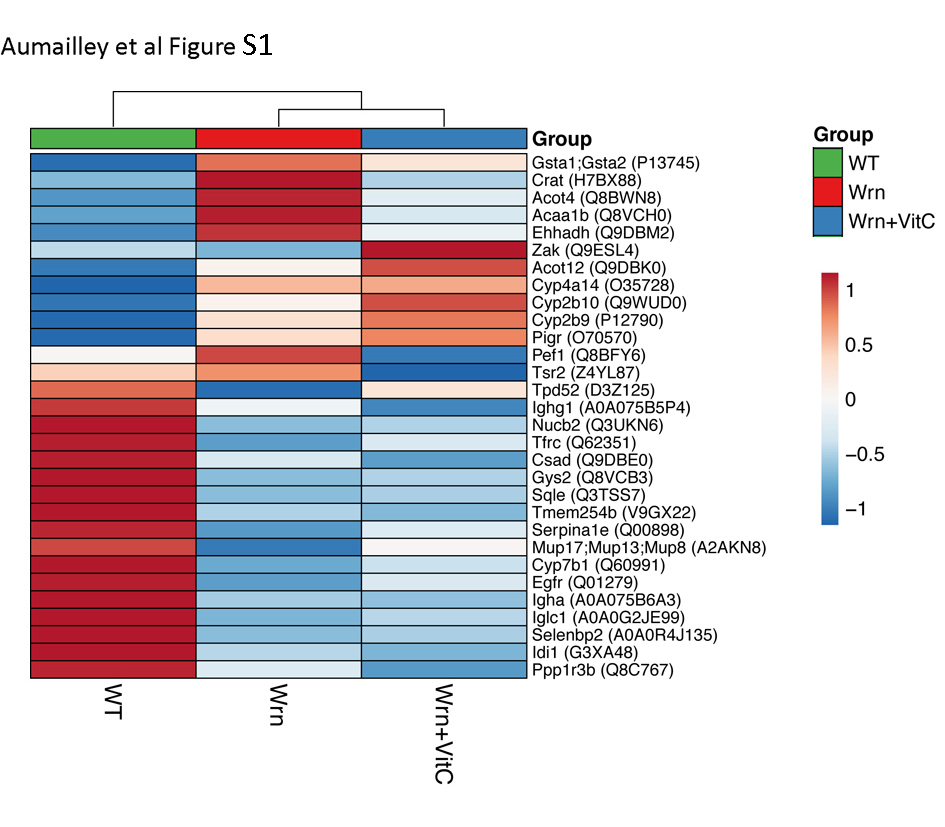

Supplement: S1 Fig — Heatmap depicting the Z-score of log base ten of the means normalized intensities of each protein (rows) between groups of mice (columns). (TIF) [file pone.0193170.s001.tif]
